# Prognostic value of Geriatric Nutritional Risk Index and systemic immune-inflammatory index in elderly patients with acute coronary syndromes

**DOI:** 10.1038/s41598-024-53540-z

**Published:** 2024-02-07

**Authors:** Xing-Yu Zhu, Kai-Jie Zhang, Xiao Li, Fei-Fei Su, Jian-Wei Tian

**Affiliations:** 1 Graduate School of Hebei North University, Zhangjiakou, 075031 Hebei Province China; 2 Department of Cardiovascular Medicine, Air Force Medical Center, Chinese People’s Liberation Army, Beijing, 100142 Beijing China

**Keywords:** Interventional cardiology, Predictive markers, Acute coronary syndromes

## Abstract

The objective of this study was to evaluate the predictive value of the Geriatric Nutritional Risk Index (GNRI) combined with the Systemic Immunoinflammatory Index (SII) for the risk of major adverse cardiovascular events (MACE) following percutaneous coronary intervention in elderly patients with acute coronary syndrome (ACS). We retrospectively reviewed the medical records of 1202 elderly patients with acute coronary syndromes divided into MACE and non-MACE groups according to whether they had a MACE. The sensitivity analysis utilized advanced machine learning algorithms to preliminarily identify the critical role of GNRI versus SII in predicting MACE risk. We conducted a detailed analysis using a restricted cubic spline approach to investigate the nonlinear relationship between GNRI, SII, and MACE risk further. We constructed a clinical prediction model based on three key factors: GNRI, SII, and Age. To validate the accuracy and usefulness of this model, we compared it to the widely used GRACE score using subject work and recall curves. Additionally, we compared the predictive value of models and GRACE scores in assessing the risk of MACE using the Integrated Discriminant Improvement Index (IDI) and the Net Reclassification Index (NRI). This study included 827 patients. The GNRI scores were lower in the MACE group than in the non-MACE group, while the SII scores were higher in the MACE group (*P* < 0.001). The multifactorial analysis revealed a low GNRI (OR = 2.863, 95% CI: 2.026–4.047, *P* = 0.001), High SII (OR = 3.102, 95% CI: 2.213–4.348, *P* = 0.001). The area under the curve (AUC) for the predictive model was 0.778 (95% CI: 0.744–0.813, *P* = 0.001), while the AUC for the GRACE score was 0.744 (95% CI: 0.708–0.779, *P* = 0.001). NRI was calculated to be 0.5569, with NRI + at 0.1860 and NRI- at 0.3708. The IDI was found to be 0.0571, with a *P*-value of less than 0.001. These results suggest that the newly developed prediction model is more suitable for use with the population in this study than the GRACE score. The model constructed using GNRI and SII demonstrated good standardization and clinical impact, as evidenced by the standard, DCA, and clinical impact curves. The study shows that combining GNRI and SII can be a simple, cost-effective, and valuable way to predict the risk of MACE within one year in elderly acute coronary syndromes.

## Introduction

Acute Coronary Syndrome (ACS) is a significant cause of mortality^1^. Despite ongoing breakthroughs in medical advances, such as percutaneous coronary intervention (PCI), a considerable number of patients, particularly in the elderly population, continue to experience major adverse cardiovascular events (MACE) each year^2–4^. Early risk stratification of patients at high risk of future MACE is essential. However, reliable indicators to predict the risk of MACE in elderly patients with ACS are still lacking.

There is increasing evidence to suggest that malnutrition and inflammation play a significant role in determining the prognosis of cardiovascular disease (CVD)^5,6^. The Geriatric Nutritional Risk Index (GNRI) was first defined in 2005 by Bouillanne et al.^7^. It can be easily calculated from routine hematological data, such as serum albumin and anthropometric data, including height and weight^7^. GNRI is a widely used nutritional indicator to predict the prognosis of cardiovascular diseases, such as heart failure and non-ST-segment elevation acute coronary syndrome, in the elderly^8,9^. The Systemic Immunoinflammatory Index (SII) is a new inflammatory index that calculates the levels of neutrophils, platelets, and lymphocytes in peripheral blood. These levels can represent various inflammatory and immune pathways in the body with greater stability^10^. SII is linked to adverse outcomes in patients with cardiovascular disease, such as atrial fibrillation, chronic heart failure, and cardiomyopathy^11–13^. Platelets and leukocytes are significant contributors to the development of atherosclerosis and acute coronary syndromes. Higher platelet counts may indicate destructive inflammatory processes and prothrombotic states^14^. Research has demonstrated that low blood lymphocyte counts are linked to more severe cardiovascular outcomes in patients with coronary artery disease (CAD)^15^. Previous studies have commonly used inflammatory hematological ratios, such as neutrophil–lymphocyte ratio, platelet-lymphocyte ratio, monocyte-lymphocyte ratio, and lymphocyte-monocyte ratio, to evaluate the prognosis of acute coronary syndromes^16,17^. However, there are limited studies on early risk stratification using SII in combination with GNRI in elderly patients at high risk of major adverse cardiovascular events after PCI for acute coronary syndromes.

Both SII and GNRI were found to be independent predictors of cardiovascular disease severity and prognosis. This enables early categorization and prevention of complications. The objective of this study was to assess the effectiveness of combining SII and GNRI for early risk stratification of elderly patients with acute coronary syndromes who are at high risk of MACE within one year.

## Methods

### Patients and participants

This retrospective observational study is based on the Chinese People's Liberation Army Air Force Medical Centre. It includes 1202 consecutive patients admitted to the cardiovascular medicine department diagnosed with ACS from August 2019 to August 2022. Based on the specified inclusion and exclusion criteria, eight hundred twenty-seven patients were included. This study included patients aged 60 or older who underwent PCI for acute coronary syndromes. ACS Definitions According to the 2018 Diagnostic Guidelines: 'Fourth Universal Definition of Myocardial Infarction (2018)^18^. In this study, patients were defined as having recurrent angina, severe arrhythmia, rehospitalization for cardiovascular reasons, acute myocardial infarction, heart failure, and death from coronary heart disease as major adverse cardiovascular events(MACE). This study excluded 286 patients due to missing clinical data. Additionally, 63 patients with hematological diseases and malignant tumors and 26 patients with missing follow-up data were also excluded. The groups were classified based on the occurrence of MACE within one year, distinguishing between those who experienced MACE and those who did not. The study adhered to the principles outlined in the Declaration of Helsinki and received approval from the Ethics Committee of the Chinese People's Liberation Army Air Force Centre. Informed consent for this study was obtained from all subjects and their legal guardians.

### Clinical and demographic characteristics

The patients' essential clinical characteristics were recorded, including Age, sex, height, weight, admission blood pressure, history of smoking, history of alcohol consumption, and presence of diabetes mellitus. Laboratory information was collected, including tests for white blood cells, neutrophils, lymphocytes, hemoglobin, platelets, ultrasensitive C-reactive protein, and lipids. Cardiac ultrasound and coronary angiography results were also collected in this study. The GNRI was calculated using the following formula: GNRI = [1.489 × albumin (g/L)] + [41.7 × (weight/WLo)]^7^. This formula was derived by substituting the ideal weights in the NRI formula with the usual weights calculated according to the Lorenz formula^7^. The Systemic Immune-Inflammation Index (SII) was calculated using the formula SII = platelets multiplied by neutrophils divided by lymphocytes. GRACE scores were calculated using the methods previously reported in the literature^19^.

### Follow-up and clinical endpoints

Experienced follow-up specialists were appointed to conduct a systematic survey to determine the presence or absence of major adverse cardiovascular events within one year after the patient's discharge from the hospital. The occurrence of such events was documented in detail.

### Statistical methods

Statistical analyses were performed using R version 4.2.3, SPSS version 27.0, and Python version 3.12 software. Firstly, Gpower 3.1.9.7 software was used to calculate the sample size required for this study, and it was found that 324 samples were required. This study contains 827 samples, which allows for a more accurate analysis of the relationship between the variables and the dependent variable. Normality tests were conducted during the preliminary stage of data analysis. Continuous variables that follow a normal distribution were described using the mean and standard deviation (*x̄* ± *s*). Independent samples t-tests were used to compare parameter values between groups. For continuous variables that do not follow a normal distribution, the median (M(Q1, Q3)) was used to express them, and the Mann–Whitney U-test was used for comparative analyses. Categorical variables were presented visually using percentages, and the differences were explored using the χ2 test. The correlation between MACE and the variables of interest was analyzed using Pearson correlation. The effects of variable feature importance and features were investigated using SHAP summary plots from the Random Forest algorithm. The study used multifactor logistic regression to examine the association between GNRI, SII, and MACE risk.

Additionally, the study used a restricted cubic spline to analyze the nonlinear relationship between GNRI, SII, and MACE risk further. Clinical prediction models and nomograms can be constructed using GNRI, SII, and Age. The Nomogram's performance was evaluated using the Hosmer–Lemeshow test to assess the goodness of fit of the model. The resulting chi-squared statistic and *P*-value were then calculated. Model discrimination was evaluated by plotting calibration curves and calculating the area under the curve (AUC) of subject operating characteristics (ROC). The validity and clinical impact of the model were evaluated using decision curve analysis (DCA) and clinical impact curves (CIC). Differences were considered statistically significant at *P* < 0.05. Additionally, we compared the predictive value of models and GRACE scores in assessing the risk of MACE by using the Integrated Discriminant Improvement Index (IDI) and the Net Reclassification Index (NRI).

## Results

### Comparison of general clinical data between MACE and non-MACE groups

Out of the 827 patients who were enrolled, 258 experienced a MACE event. The patients were divided into MACE and non-MACE groups based on whether they experienced a MACE within one year. Table 1 summarises the baseline characteristics of the MACE and non-MACE groups. There were no statistically significant differences between the MACE and non-MACE groups regarding gender, high-density lipoprotein, platelet count, and prevalence of diabetes mellitus (*P* > 0.05). However, the median Age in the MACE group was significantly higher at 69 years compared to 65 years in the non-MACE group. The mean GNRI score was 107.00 in the MACE group and 113.39 in the non-MACE group. The median SII score was 868.84 in the MACE group and 548.20 in the non-MACE group. On average, the mean GRACE score was 112.35 in the MACE group and 95.94 in the non-MACE group.

**Table 1:** Comparison of general clinical data between MACE and non-MACE groups. MACEs: major adverse cardiovascular events; BMI: body mass index; LVEF: left ventricular ejection fraction; GRACE: global registry of acute coronary events.

Characteristic	Non-MACE (n = 569)	MACE (n = 258)	*P*-value
Age[years, M (Q_1_, Q_3_)]	65 (61, 69)	69 (65, 76)	0.001
Sex, male, n (%)	451 (79.26)	194 (75.1)	0.191
Smoking, n (%)	195 (34.27)	107 (41.47)	0.046
Diabetes, n (%)	232 (40.77)	111 (43.02)	0.543
Hypertension, n (%)	377 (66.25)	184 (71.32)	0.149
Dyslipidemia, n (%)	351 (61.69)	142 (55.04)	0.071
White blood cell count [/10^9^L^−1^, M (Q_1_, Q_3_)]	6.95 (5.68, 8.50)	7.39 (5.64, 10.52)	0.039
Neutrophils [/10^9^L^−1^, M (Q_1_, Q_3_)]	4.37 (3.36, 5.45)	5.77 (4.30, 8.53)	0.001
Lymphocytes [/10^9^L^−1^, M (Q_1_, Q_3_)]	1.66 (1.27, 2.08)	1.35 (0.97, 1.84)	0.001
Hemoglobin [g/L, [*x̄* ± *s*]]	140.57 ± 18.76	129.73 ± 24.10	0.001
Creatinine [umol/L, [*x̄* ± *s*]]	351.13 ± 97.81	367.30 ± 98.13	0.001
Uric acid [umol/L, M (Q_1_, Q_3_)]	342.00 (286.00, 396.00)	353.00 (296.00, 433.00)	0.029
Albumin [*x̄* ± *s*]	43.39 ± 3.34	39.82 ± 3.53	0.001
Myoglobin [ng/mL, M (Q_1_, Q_3_)]	51.00 (27.60, 74.00)	76.00 (45.00, 238.00)	0.001
Creatine kinase isoenzyme [ng/mL, M (Q_1_, Q_3_)]	2.00 (2.00, 4.00)	2.90 (2.00, 30.60)	0.003
Cardiac troponin [ng/mL, M (Q_1_, Q_3_)]	0.01 (0.01, 2.18)	0.16 (0.01, 6.00)	0.001
Total cholesterol [*x̄* ± *s*]	4.28 ± 1.28	3.91 ± 1.03	0.001
Triglyceride [mmol/L,M (Q_1_, Q_3_)]	1.40 (1.04, 2.00)	1.28 (0.99, 1.73)	0.039
High-density lipoprotein [*x̄* ± *s*]	1.05 ± 0.24	1.03 ± 0.24	0.353
Low-density lipoprotein [mmol/L,M (Q_1_, Q_3_)]	2.09 (1.59, 2.67)	2.33 (1.71, 2.88)	0.009
NT-proBNP [pg/mL, M (Q_1_, Q_3_)]	49.40 (18.60, 108.30)	151.90 (56.80, 528.20)	0.001
BMI [*x̄* ± *s*]	25.49 ± 3.66	24.9 ± 3.38	0.016
LVEF%[*x̄* ± *s*]	60.45 ± 5.53	53.89 ± 7.18	0.001
GNRI [*x̄* ± *s*]	113.39 ± 9.07	107.00 ± 8.59	0.001
SII [M (Q_1_, Q_3_)]	548.20 (377.83, 774.13)	868.84 (536.73, 1614.12)	0.001
GRACE [*x̄* ± *s*]	95.94 ± 16.22	112.35 ± 19.06	< 0.001

**Figure 1:**
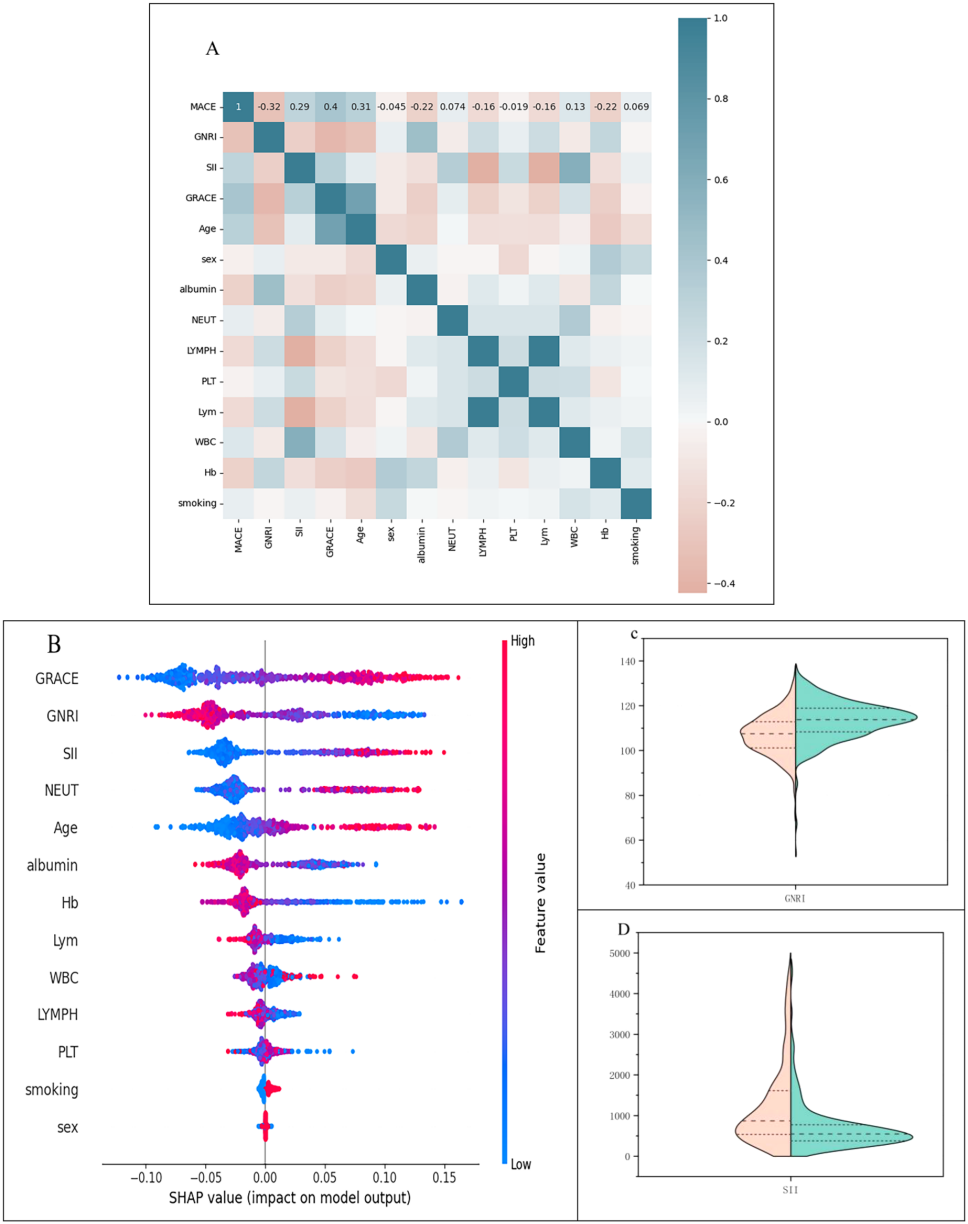
(**A**) The Pearson analysis of MACE in acute coronary syndromes in the elderly and related factors. (**B**) SHAP Summary Chart. Application of machine learning to feature selection. The features are ordered on the vertical axis based on the sum of their SHAP values across all samples. The horizontal axis represents the distribution of the effects of the features on the model output. Each point on the graph represents a sample, with the sample size stacked vertically. The colors indicate the eigenvalues, with red representing high values and blue representing low values. (**C**, **D**) Distribution of GNRI and SII in various subgroups. The MACE group is represented by the pink area, while the non-MACE group is represented by the green area. Each region is represented by three dotted lines indicating the 25th, 50th, and 75th quartiles. The violin plot's wider section indicates a higher probability of an observation taking a particular value, while the narrower section corresponds to a lower probability.

A Pearson analysis was conducted to examine the occurrence of MACE in the year following PCI for acute coronary syndromes in elderly patients concerning various clinically relevant parameters. The results showed that MACE was significantly correlated with Grace, GNRI, and SII. The SHAP summary plot also further confirmed the significant association of MACE occurrence with Grace, GNRI, and SII. The violin plot shows that patients in the MACE group had lower GNRI levels and higher SII levels (Fig. 1).

### Analysis of risk factors for MACE

**Figure 2:**
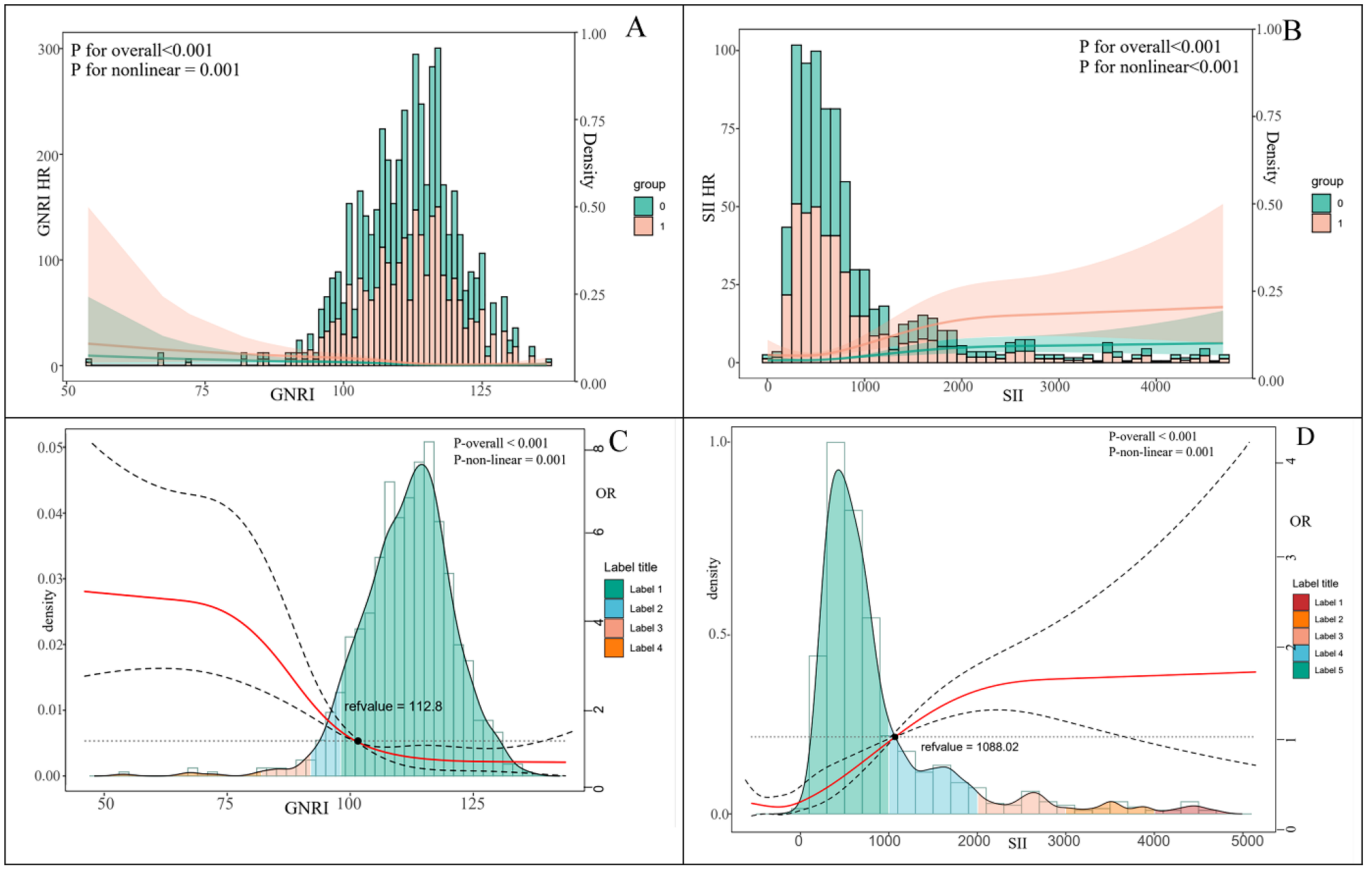
Restricted cubic spline relationships between GNRI, SII, and MACE risk. 1 in Group grouping in Figures (**A** and **B)** represents Age greater than or equal to 68, and 0 represents less than 68. Multivariable adjusted risk ratios for MACE risk ratios adjusted for SII and GNRI scores on continuous scales. The red solid line is the multivariate-adjusted risk ratio, and the dashed line is the 95 percent confidence interval derived from a three-section restricted cubic spline regression. A dashed line with a risk ratio of 1.0 indicates the unrelated reference line. The sizes of GNRI and SII are shown separately on the x-axis. The distribution of GNRI and SII score densities in the study population is presented in histograms and density plots.

ROC curves were utilized to analyze the capacity of GNRI, SII, and Age to identify post-PCI MACE in elderly ACS patients. Each of the variables, GNRI, SII, and Age, was divided into two groups based on the optimal threshold. The optimal cut-off value for SII is 753.93, and for GNRI, it is 111.00^20,21^. As for Age, the best cut-off value is 68. One-way logistic regression was used to analyze the GRACE scores, with MACE occurrence as the endpoint indicator. The study conducted multifactor logistic regression analyses on SII, GNRI, and Age. In regression analysis, a positive regression coefficient and an odds ratio (OR) greater than 1 indicate a risk factor that threatens the outcome. Conversely, a negative regression coefficient and an OR less than 1 indicate a protective factor that safeguards the outcome. Table 2 presents the risk factors, which include high SII, low GNRI, high Age, and GRACE. The study evaluated the association between GNRI, SII, and MACE risk using a multifactor logistic regression model with restricted cubic splines. The study evaluated the association among GNRI, SII, and MACE risk by using restrictive cubic sample bars. GNRI, SII, and risk of MACE were nonlinearly correlated (P-non-linear = 0.001). The relationship between GNRI, SII, and MACE is more intuitively represented by the restrictive cubic spline in Fig. 2, where the lines trend downward as the GNRI score increases and upward as the SII score increases.

**Table 2:** Results of one-factor and multifactor logistic regression analyses of mace. β: regression coefficient; OR: Odds Ratios, S.E.: Standard Error.

Characteristic	β	SE	Wald	*P* value	OR	95% CI
High SII	1.132	0.172	43.134	0.001	3.046	2.182–4.251
Low GNRI	1.052	0.177	35.502	0.001	3.222	2.296–4.523
GRACE	0.028	0.007	16.975	0.001	1.051	1.041–1.060
High Age	0.876	0.175	25.177	0.001	2.401	1.705–3.380

### Construction and evaluation of MACE predictive models

**Figure 3:**
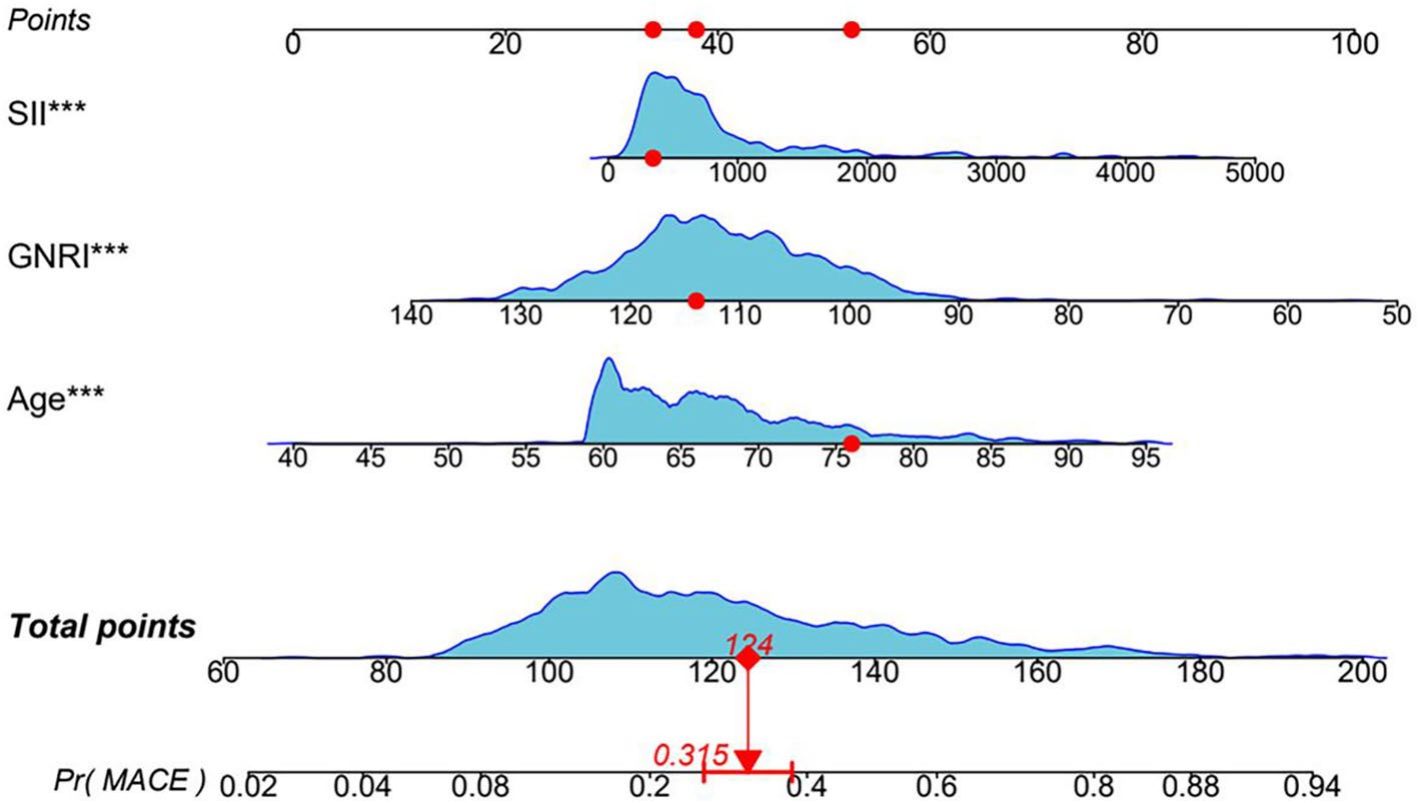
Nomogram of the clinical prediction model for the occurrence of MACE within one year after PCI in elderly ACS patients.

The three factors in the multifactorial logistic regression model were used as predictors to construct a column-line diagram of the clinical prediction model for the occurrence of MACE within one year after PCI in elderly ACS patients, as shown in Fig. 3. The three factors in the multifactorial logistic regression model were used as predictors to construct a nomogram of the clinical prediction model for the occurrence of MACE within one year after PCI in elderly ACS patients, as shown in Fig. 3. For each patient, each identified risk factor or clinical characteristic was scored, and the total score was the sum of the scores for each variable calculated from the Nomogram corresponding to the predicted value of MACE at one year. The results of one of the cases showed that the sum of the scores of the variables SII, GNRI, and Age was 124, and the probability of risk of MACE within one year in this patient was approximately 31.5%, with a 95% confidence interval of 25.9–37.8%.

### Comparison of clinical predictive modelling and GRACE scores

The Net Reclassification Index (NRI) is a vital tool for assessing the accuracy of predictive models. If the NRI is greater than 0, it usually indicates that the new model is outperforming the old model in terms of predictive performance; if the NRI is less than 0, it suggests that the new model is underperforming compared to the old model. The Integrated Discriminant Improvement Index (IDI) focuses on the change in the difference in predicted probability between the two models based on the expected probability of each individual in the disease model. Overall, a higher value of the IDI means that the new model has a greater degree of superiority in its predictive ability. If the IDI is more significant than zero, it indicates an improvement; if the IDI is less than zero, it shows a negative improvement; and if the IDI is equal to zero, it means that the new model has not brought any improvement. NRI represents the net reclassification index for the data as a whole; NRI + refers specifically to the net reclassification index in the data where a MACE event occurred; and NRI- indicates the net reclassification index in the data where no MACE event occurred. The results were NRI = 0.5569; NRI +  = 0.1860; NRI- = 0.3708; IDI = 0.0571, and 95% confidence intervals not including 0, with a *P*-value < 0.001, as shown in Table 3. It is suggested that the newly developed prediction model is more suitable than the GRACE score for use with the population in this study.

**Table 3:** Comparison of clinical prediction models and GRACE scores.

	Estimate	S.E.	95% CI	*P* value
NRI	0.5569	0.0787	0.3803–0.6955	< 0.001
NRI+	0.1860	0.0693	0.0350–0.2984	< 0.001
NRI−	0.3708	0.055	0.2635–0.4797	< 0.001
IDI	0.0571	NA	0.0395–0.0746	< 0.001

**Figure 4:**
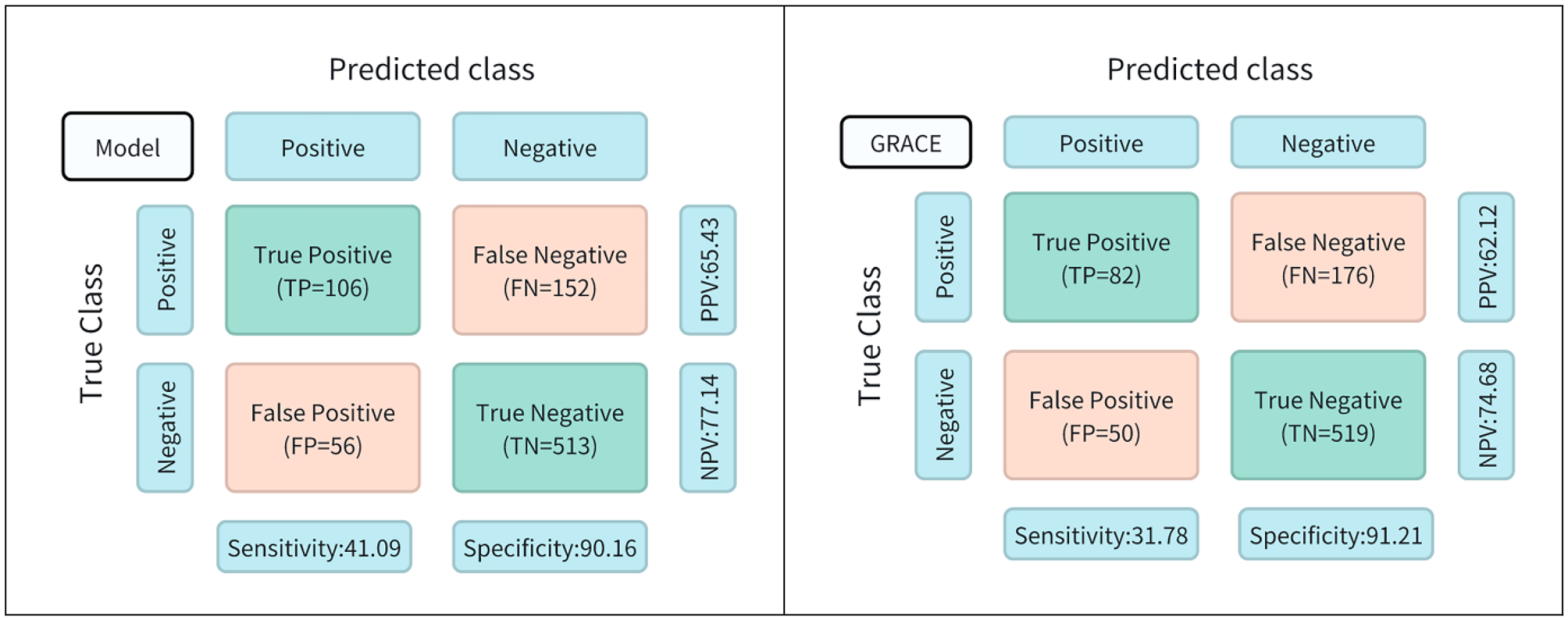
Efficacy of Predictive Models and GRCE Scores for Assessing MACE Risks.

**Figure 5:**
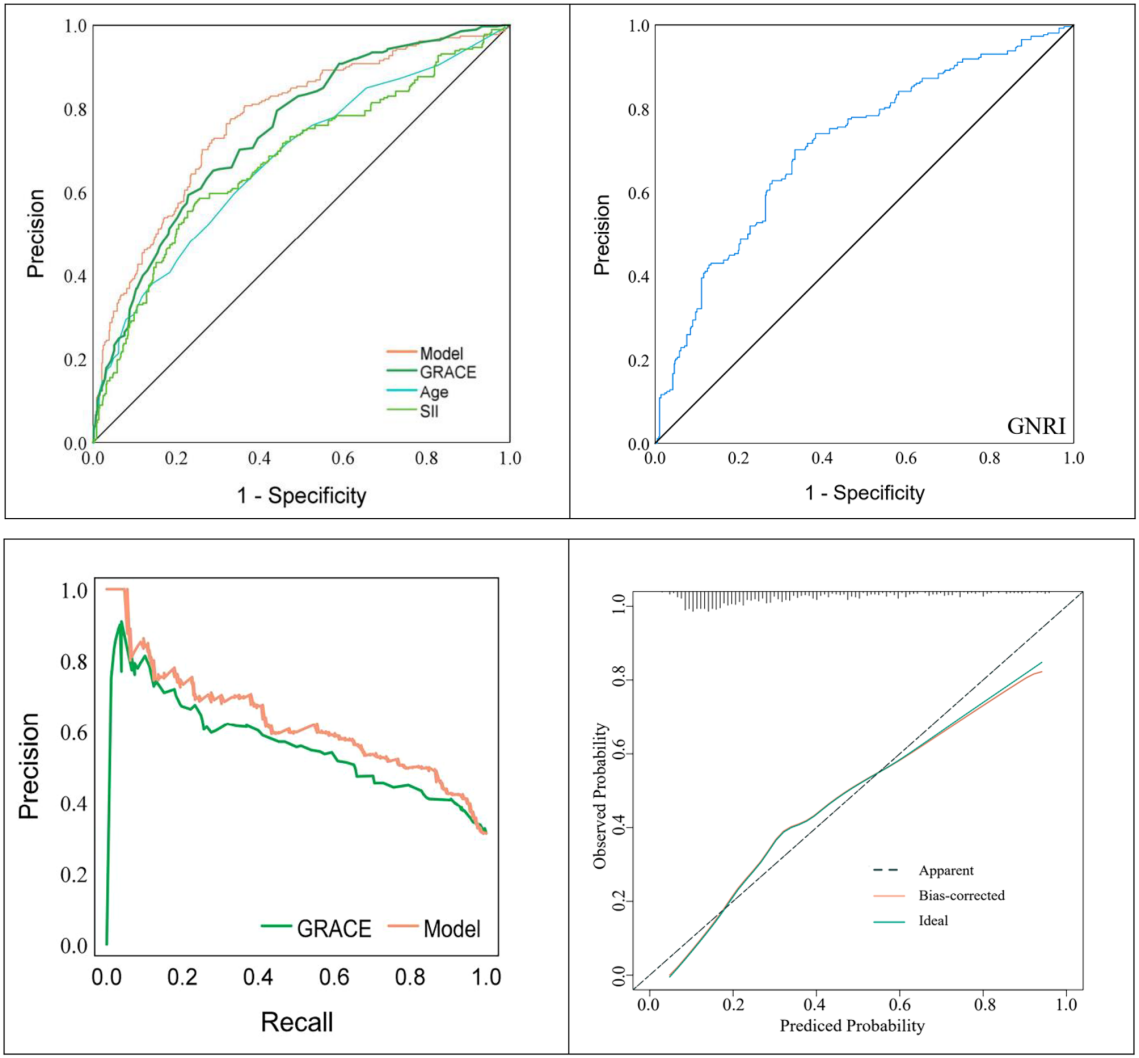
ROC and recall curves were used to predict MACE in elderly ACS patients within one year of PCI. Standard Curve for Clinical Prediction Model for the Development of MACE within One Year of PCI in Elderly Patients with ACS.

Sensitivity, specificity, positive predictive value (PPV), and negative predictive value (NPV) were calculated for the model, and the GRACE score was used to assess the risk of MACE. As shown in Fig. 4, the sensitivity of the predictive model 41.09, NPV 77.14, and PPV 65.43 were higher than the GRACE score. In this study, the ROC curve analysis of the prediction model for the occurrence of MACE within one year in elderly ACS patients undergoing PCI is shown in Fig. 5. The AUC of the prediction model = 0.778, 95% CI: 0.744–0.813, *P* value 0.001; the AUC of the GRACE score = 0.744, 95% CI: 0.708–0.779,* P* value 0.001(Table 4). As shown by the recall curve in Fig. 5, the recall of the predictive model is more favorable than that of GRACE. The horizontal coordinate of the calibration curve is the prediction probability of the model, and the vertical coordinate is the probability of the observation in the actual data. According to the calibration curve, the prediction model constructed in this study using SII, GNRI, and Age has a good calibration ability (Fig. 5).

**Table 4:** Area under the ROC curve.

	AUC	*P*	95% CI	Youden index	Best cut-off value
GNRI	0.712	0.001	0.674–0.750	0.368	111.00
SII	0.676	0.001	0.635–0.717	0.330	753.93
Age	0.675	0.001	0.635–0.716	0.258	68
GRACE	0.744	0.001	0.708–0.779	0.361	104.50
Model	0.778	0.001	0.744–0.813	0.445	0.26

**Figure 6:**
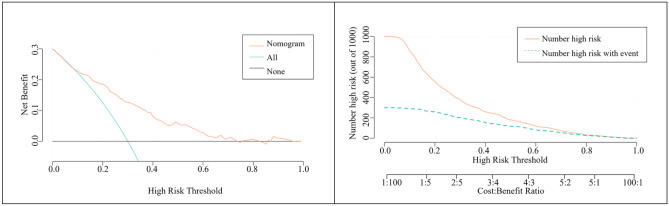
DCA Curve and Clinical Impact Curve of a Clinical Prediction Model for the Development of MACE within One Year of PCI in Elderly Patients with ACS.

In Fig. 6 DCA curve, there is a black line, a green line, and a red line; the black line is if all people are not treated, then the net benefit of treatment must be 0. The green line is if all people are treated, then the value decreases as the threshold probability increases. The graph shows the threshold probability versus net benefit for the decision model. A model is considered to have no application value if the red line is close to the black and blue reference lines. Conversely, a model is considered to be better if the red line is above the reference lines for a large threshold interval. According to the decision-analysis curves (DCA), the use of the predictive model to estimate the risk of MACE one year after PCI in elderly ACS patients was more favorable than implementing an intervention program for all patients when the threshold probability exceeded 15%. The net benefit of the predictive model was also significantly higher than that of an all-or-none intervention (Fig. 6). Clinical impact curves (CIC) were plotted based on DCA to assess the clinical impact of each model. The curves show the estimated number of people predicted to have MACE and the actual number of people with the disease at each risk threshold. Figure 6 demonstrates that the clinical prediction models we constructed had a positive clinical impact.

## Discussion

Acute coronary syndrome (ACS) is caused by the rupture of unstable atherosclerotic plaques in the coronary arteries. This occurs as a result of a chronic inflammatory response of leukocytes in the arterial wall^22^. Malnutrition is linked to inflammation, which leads to an increased burden of atherosclerosis. The relationship between these three factors has been recently described as the malnutrition-inflammation-atherosclerosis syndrome^23,24^. Although there have been significant advances in diagnosing and treating acute coronary syndromes in recent decades, cardiovascular disease remains the leading cause of death worldwide. Ischaemic heart disease is responsible for almost half of these deaths^25^. Older adults with increased atherosclerotic plaque burden and complex anatomical disease, along with age-related cardiovascular and non-cardiovascular comorbidities, have a worse prognosis^4,26^. Patients with acute coronary syndromes may also experience cognitive dysfunction and frailty^27,28^. Early identification of patients at high risk of future adverse cardiovascular events is essential. As far as we know, no research has investigated the use of GNRI combined with SII score to predict the risk of major adverse cardiovascular events within one year after PCI for acute coronary syndromes in elderly patients. This study is the first to use the GNRI combined with the SII score to predict the risk of major adverse cardiovascular events within one year after PCI for acute coronary syndromes in the elderly.

Recent studies have found a correlation between malnutrition and all-cause mortality, as well as MACE^5,29^. Tonet et al. reported that 44% of elderly patients with ACS suffered from malnutrition or were at risk of malnutrition and that malnutrition is an independent and strong risk factor for all-cause mortality in elderly patients with ACS^30^. Low levels of albumin have been shown to have prognostic value in both coronary artery disease and stable coronary artery disease^31^. Suzuki et al.'s study concluded that atherosclerotic systemic inflammation causes low albumin levels in patients with stable coronary artery disease (CAD)^32^. The GNRI is a readily available indicator that combines serum albumin levels and body weight. It is often used to assess the nutritional status of older people who are hospitalized^33^. Therefore, this study used GNRI to evaluate the risk of adverse cardiovascular events within one year after PCI in elderly patients with acute coronary syndromes. The study found that the GNRI score in the MACE group was significantly lower than in the non-MACE group. The violin plot shows the distribution of GNRI scores for the two subgroups. The GNRI scores in the MACE group were lower than those in the non-MACE group. The SHAP summary plot indicates a significant correlation between GNRI and the risk of MACE. In summary, a lower GNRI score is associated with a higher risk of MACE, which is consistent with Yoo et al.'s study^34^.

Inflammation plays a crucial role in the formation of blood clots^35,36^. Neutrophils can be detected in atheromatous plaques and may contribute to plaque formation and increased blood clot stability^37^. Zhang and colleagues found that the neutrophil count independently influenced the occurrence of major adverse cardiovascular events (MACE) in patients with ST-elevation myocardial infarction (STEMI)^38^. Platelets play a crucial role in thrombosis and significantly affect the pathogenesis of atherosclerosis and the development of acute thrombotic coronary events^39^. Elevated platelet counts may indicate an increase in the release of inflammatory mediators, leading to heightened platelet activation, which can trigger a destructive inflammatory response and the emergence of a prothrombotic state. Recent studies have demonstrated that acute STEMI patients with elevated platelet counts have a poorer prognosis^40^. Lower lymphocyte counts are associated with an increased risk of cardiovascular disease and mortality^41,42^. Therefore, it is essential to maintain healthy lymphocyte levels. To create a more stable inflammation index, researchers have proposed the Systemic Immune Inflammation Index (SII). This new index is based on the calculation of peripheral blood neutrophils, platelets, and lymphocytes and has been widely used to predict the prognosis of diseases such as tumors, cardiovascular diseases, and cerebrovascular diseases^10,43^. In a recent study, Geng and colleagues found that the Systemic Immune-Inflammation Index (SII) has a better prognostic value than the Ratio of Neutrophils to Lymphocytes (RNL) and the Ratio of Platelets to Lymphocytes (RPL)^44^. SII is a new index that measures inflammation. It is strongly linked to cardiovascular and all-cause mortality. To improve prevention strategies, more attention should be given to systemic inflammation^45^. Table 1 and Fig. 2 show that the SII score was significantly higher in the MACE group than in the non-MACE group. The Pearson correlation analysis and the SHAP summary graph visualize a significant correlation between the SII score and the risk of MACE within one year after PCI for acute coronary syndromes in the elderly.

The study used GNRI combined with SII score to predict the risk of MACE within one year after PCI in elderly patients with acute coronary syndromes. We created a clinical prediction model using GNRI, SII, and Age and compared it to the GRACE score. The study results indicate that the prediction model has an excellent discriminatory ability, with an AUC of 0.778 compared to the GRACE score's AUC of 0.744. The PRECISION-recall curve shows that the prediction model has a larger area under the curve than the GRACE score. This indicates that the clinical prediction model we constructed has good recognition accuracy. Following calculations, we obtained the sensitivity, specificity, positive predictive value, and negative predictive value. The results indicate that the clinical prediction model outperformed the GRACE score in terms of sensitivity, NPV, and PPV. The standard curve demonstrates that the model is more accurate. Additionally, the decision analysis curve and the clinical impact curve show satisfactory net benefits from the model.

While this study is the first to confirm the effectiveness of the GNRI combined with the SII score in predicting the risk of MACE after PCI for acute coronary syndromes in the elderly, its retrospective single-center design remains a limitation. A more in-depth understanding of the dynamics of the GNRI and SII scores would allow for a more accurate assessment of the association between these metrics and MACE risk. It is expected that this study will provide a solid foundation for future prospective multicentre studies.

## Conclusion

The combination of the GNRI score and the SII score is a reliable, simple, cost-effective, and easily accessible metric for predicting the risk of MACE in elderly patients undergoing PCI for acute coronary syndromes. This helps cardiovascular specialists to stratify patients' risks and reduce the incidence of MACE.

## Data Availability

The datasets used and/or analyzed during the current study are available from the corresponding author upon reasonable request.

## Abbreviations

ACS: Acute coronary syndromes
PCI: Percutaneous coronary intervention
MACE: Major adverse cardiovascular event
GNRI: Geriatric Nutritional Risk Index
SII: Systemic Immune Inflammatory Index
GRACE: Global registry of acute coronary events
